# Prioritizing ethical patient care responsibilities: A call to restructure student training practices in federally qualified health centers

**DOI:** 10.1016/j.rcsop.2023.100320

**Published:** 2023-08-21

**Authors:** Natalie Rosario, Joshua Wollen, Edward D. Scott

**Affiliations:** a Pharmacy Practice and Translational Research, Health 2, University of Houston College of Pharmacy, 4349 Martin Luther King Boulevard, Houston, TX 77204-5039, United States of America; b Social Work, University of Houston, 3511 Cullen Blvd., Houston, TX 77204, United States of America

**Keywords:** FQHC, Clinical rotations, APPE, Medically underserved, Ethical care

## Abstract

Federally Qualified Health Centers (FQHCs) are federally funded clinics that often serve medically underserved groups. Many Colleges of Pharmacy have faculty and non-faculty pharmacist preceptors who provide clinical services such as drug therapy management to FQHCs. It is critical that Colleges of Pharmacy and pharmacist preceptors reinforce and uphold the standard of providing high quality and evidenced based care when students rotate at these sites. Learners may have implicit biases and variable levels of emotional intelligence prior to a clinical rotation at an FQHC, which can affect the quality-of-care patients receive. Colleges of Pharmacy who send learners onto rotations at FQHCs should collaborate with FQHC sites to ensure learner readiness in clinical and emotional levels and mediate for any concerns that may arise.

## Highlights

• FQHCs are federally funded clinics that serve medically underserved groups.
• FQHCs employ or contract ambulatory care pharmacists to provide clinical services, often in collaboration with colleges of pharmacy.
• There is a lack of data showing improvement in patient outcomes when pharmacy trainees at FQHCs are involved in patient care.
• It is critical that the quality of patient care is not scarified for trainee learning at the expense of patients' health.

## 1 Characteristics of federally qualified health centers

The Federally Qualified Health Center (FQHC) has become an important resource for providing outpatient healthcare for socioeconomically disadvantaged individuals in the United States. Patients receiving care from an FQHC may be eligible for discounted rates on services and cannot be denied care regardless of their ability to pay.^1^ FQHCs are also required to have a board comprised of at least 51% patients.^2^^,^^3^ They are funded by state and federal grants, local foundations, and community funding.^4^ Third party payments are made to the clinic through private insurance, Medicare, Medicaid, or the Children's Health Insurance Program (CHIP). For patients without insurance, the clinic service fees are determined by family size and/or income via copayments, sliding fee scale payment, or direct cash payments.^3^

Additionally, pharmacy services that may be offered by FQHC pharmacists include Medication Assistance Programs (MAP) and 340B drug pricing programs.^5^^,^^6^ MAP is a program where patients who do not have prescription insurance and meet financial thresholds can apply to receive free medication from the drug manufacturing company. Some drug manufacturers also require that the applicant is a US citizen or can provide an Individual Taxpayer Identification Number. MAP are often utilized for medications that are brand name and expensive. Another medication cost-savings program that FQHCs may utilize includes 340B drug pricing where specific pharmacies have contracts with drug manufacturers for highly discounted prices on brand and generic medications for individuals who do not have prescription drug insurance. For patients with prescription drug insurance, the healthcare team may submit a prior authorization (PA) to the insurance company providing rationale as to why a patient may need to utilize a more costly medication that is not listed on the insurance's preferred drug list. The PA process is not unique to FQHCs but is another means to provide patients with more affordable medication options.

The Uniform Data System (UDS) reports standardized information regarding health centers that serve underserved and vulnerable communities in the United States. The most recent data available from the National Health Center Program UDS in 2021 reported demographic patient characteristics for 30,193,278 FQHC patients. Medicaid and CHIP accounted for 48% of the patients' third-party payers, 20% had no insurance, 20% had private insurance, and 10% had Medicare. Sixty-seven percent of patients' income was below the federal poverty guideline (33% had unknown income) and 10% of patients' income was over 200% of the poverty guideline. Racially and ethnically marginalized patients (Black, Hispanic or Latine, Indigenous, or Pacific Islander) accounted for 61% of FQHC patients.^5^ Twenty-four percent of patients were best served in a language other than English.^7^ Patients from disadvantaged socioeconomic backgrounds are often especially vulnerable. Thus, social determinants of health (SDOH) must be extensively considered when providing therapeutic or preventative care. As healthcare professionals, pharmacists have a responsibility to prioritize patient care for such patients and treat them holistically as prescribed by the Ignatian values of “*cura personalis*,” “faith that does justice,” and “men and women (people) for and with others.”^1^

## 2 Ambulatory care pharmacists role and integration in FQHCs

Many FQHCs and clinics that serve underserved patient populations integrate ambulatory care pharmacists as full-time employees or contracted through colleges of pharmacy as a clinical practice site.^2^ The 2021 UDS data reported 1852 student pharmacists, 460 post graduate trainees, and 7095 full-time equivalents of pharmacy personnel (including pharmacists and technicians) at 1373 FQHCs in 2021 which are the most recent available data at the time of this submission.^3^

One national survey targeting ambulatory care pharmacists who work in underserved primary care areas found that most pharmacist respondents were non-Hispanic white, female, and spoke English as their only language.^2^ Respondents were identified through national professional pharmacy organization listservs such as the American College of Clinical Pharmacy, the American Society of Health-System Pharmacists, the American Pharmacists Association, and an informal listserv of clinical pharmacists self-identified to be practicing in underserved settings. Ideally, this could catch many ambulatory care pharmacists working in underserved settings if they are affiliated with national professional pharmacy organizations. When pharmacists are employed at FQHCs, they take on interprofessional and multifaceted roles that adapt to their clinical environment. For example, ambulatory care pharmacists often conduct chronic disease state management for diabetes, hypertension, dyslipidemia, thyroid disorders, behavioral health, and respiratory conditions in collaborative drug therapy management (CDTM) appointment-based visits. However, pharmacists have also spearheaded initiatives for preventive medicine (i.e., colonoscopy prep, immunizations, tobacco cessation, and falls prevention), infectious diseases like Hepatitis C and HIV, and pain management.^5^ Ambulatory care pharmacists in FQHCs may also take a population health approach.^7^^,^^8^ FQHCs have patient populations with individual cultural, language, socioeconomic, and SDOH needs. There are a variety of pharmacist-led services at FQHCs which have the potential for expanded services to enhance patient care.

Early exposure to medically underserved clinics correlated with pursuing a career as an ambulatory care pharmacist.^2^ Stewart et al. (2021) argue that colleges of pharmacy that contract faculty to underserved clinics could expose trainees to these patient populations and clinical practice sites as a learning opportunity. While this can assist with future employee recruitment, FQHCs and medically underserved clinics should approach these opportunities cautiously to ensure adequate supervision, intervention, and attention is provided to trainees to minimize the risk of patient harm. This should be approached cautiously and with patient care at the forefront. The authors feel that merely exposing trainees to underserved patients does not automatically enhance the level of care that patients receive. Student learning should not occur at the expense of a patient receiving health care services, especially given the vulnerability of patients seen at FQHCs.

Pharmacists who serve as preceptors for colleges of pharmacy are responsible for the experiential learning of final year student pharmacists. While on experiential rotations, students work under the supervision of the precepting pharmacist. Student tasks may include conducting medication reconciliation, patient interviews, informing patients about lab results, and making pharmacotherapy recommendations in conjunction with the pharmacist preceptor. As students progress through the rotation, they may be given more autonomy to complete tasks such as interviews or patient phone calls under indirect supervision, but pharmacotherapy recommendations must be made in collaboration with the pharmacist.

There are data describing burnout that clinicians at FQHCs may experience.^9^, ^10^, ^11^, ^12^ There is no direct comparison of burnout experienced by clinicians at FQHCs versus other outpatient clinic types. Major themes related to FQHC clinician burnout include limited resources, barriers in building trust with patients, administrative tasks, and compassion fatigue.^11^ These are themes that may be applicable to pharmacist clinicians as well who provide collaborative care to patients seen at FQHCs. To provide, maintain, and protect high quality patient care at FQHCs, sites must then also protect pharmacist preceptors from burnout, which could be amplified by requirements to take a particular number of trainees be it students, residents, or fellows which will be discussed further.

Additionally, pharmacists have an ethical role in patient care. The American Association of Colleges of Pharmacy in collaboration with the American Pharmacists Association have created the Oath of a Pharmacist which consists of a series of statements that student pharmacists commit to during their white coat ceremony at the beginning of their PharmD program and often at graduation as well. Among these statements are “I will consider the welfare of humanity and relief of suffering my primary concerns,” “I will apply my knowledge, experience, and skills to the best of my ability to assure optimal outcomes for my patients,” and “I will embrace and advocate changes that improve patient care.”^13^ Pharmacists have an ethical responsibility to center patients first and foremost. All clinical decisions made by pharmacists must be done weighing patient care as the implication with the most consideration.

## 3 Patient satisfaction with pharmacist-led FQHC services

There are little data regarding patient satisfaction with pharmacist-led services in FQHC settings. At one FQHC in Austin, Texas, patients rated their visits with a clinical pharmacist favorably.^14^ However, this research utilized a convenience sample of 99 patients which was acknowledged as a limitation. Patients had to read English or Spanish to participate. The clinical site had full time pharmacists, college of pharmacy faculty pharmacists, PGY2 ambulatory care pharmacy residents, and student pharmacists involved in the care of patients. However, the survey did not capture these data and the results did not distinguish ratings between these different roles. The survey did not parse out the patient's preferred language or language fluency of the pharmacist. It was found statistically significant that Hispanic patients rated their visits less favorably compared to African American (or Black) patients. There were no other differences between race or ethnicity and pharmacist rating. Language congruence between patient and pharmacist may have positively impacted the scores assigned to pharmacists by Hispanic patients. Regardless, patient satisfaction with the pharmacist and service should be taken into consideration before integrating trainees.

A survey conducted at an FQHC in Texas showed that Spanish-speaking patients preferred a Spanish-speaking pharmacist over other interpretation services including clinic staff, professional interpreters, phone interpreters, or using family or friends.^4^ This reinforces other literature that ties higher patient satisfaction to provider concordance.^15^ There is a demographic discrepancy of the current ambulatory care pharmacists providing care to underserved populations versus Spanish-speaking patient communication preferences. This highlights a disparity in care that patients who speak another language may face when seeking care at FQHCs. This discrepancy may also be present as provider discordance in other ways such as race, ethnicity, cultural, and socioeconomic background.

Simmons et al. (2019) described a diabetes education rotation that integrated student pharmacists. This study acknowledged patient concerns such as frustration or confusion surrounding the transient nature of pharmacy students through the clinic as new students rotate every 6 weeks. The workaround described is for the pharmacist to introduce themselves at the initial visit and periodically after to remind patients that they are behind the scenes as pharmacy students rotate in and out of the FQHC site, and they let patients know they are available if the patient wants to speak with them.^16^ While pharmacist interaction at each visit should be the norm, FQHC patients should not receive substandard care from trainees in place of pharmacist-led and supervised care.

## 4 Evidence for the benefits of learning experiences for pharmacy students

Some authors assert that there are beneficial impacts on student pharmacists learning when they train in FQHCs related to empathy, growth in knowledge, improvement in communication skills, and more (Table 1). Unfortunately, the patients seen at FQHCs or clinical experiences at FQHCs are often labeled “unique,” which can give the impression of being unusual or a novelty to observe. Individuals who receive services at FQHCs may have additional barriers to care and SDOH, but this should not be put on display as an exhibition or diminish the care patients receive.^16^, ^17^, ^18^ Additionally, while student learning is valuable and critical to progression through the Doctor of Pharmacy curriculum, it should not come at the expense of high quality and ethical patient care.

**Table 1:** Examples of student pharmacist experiences with underserved populations.

Practice Site	Number of Students	Duration of Experience	Year in PharmD curriculum	Patient population as described in manuscript	Description of Intervention	Description of Outcome
Two FQHCs in Indiana^15^	11	6 weeks	P4	Lack of transportation or health insurance.	Weekly reflections using the Driscoll method. Student activities included point of care INR and blood glucose testing, patient medication and lab education, medication access with MAP and PAs.	Reflections demonstrated that rotation experiences contributed to their growth and learning in communication, collaboration, and empathy. Additionally, students indicated that they gained confidence and skills.
FQHC in Colorado^22^	68 total, 37 completed pre and post surveys	6 weeks	P3 and P4	Uninsured, undocumented, limited literacy, homeless, medically complex, patients with HIV, known drug use, non-English speaking, children (ages 0–17 years), mentally compromised, and pregnant patients	Pre/post survey to evaluate students' attitudes and perceptions before and after a six-week clinical rotation experience in an underserved setting. Student activities included medication counseling, medication device teaching, and chronic disease state education and management.	Clinical rotations within an FQHC clinic can positively impact pharmacy students' attitudes towards underserved populations
Primary Care Clinic in Rural Guatemala^17^	12	6 weeks	P4	Rural Guatemalan banana plantation workers	Quantifying number of interventions (% accepted by medical team), medication counseling, QI projects, community visits with local nursing team.	Students made 1191 patient care recommendations, of which 969 were accepted (81.4%). Students completed 17 patient education and quality improvement projects.
FQHC in Abilene Texas^14^	21 total (eight P3s and 13 P4s)	6 weeks	P3 onsite 16 h per week and P4 onsite 20 h per week	Self-pay, Medicaid, Medicare, and private insurance	Face-to-face visits, active members of the interprofessional team to help improve patient outcomes. Students perform patient calls about lab results and medication reconciliations. Students make evidence- based pharmacotherapy recommendations, promote lifestyle interventions, and educate patients on self-management skills.	Site evaluations showed the opportunity to see complex and often poorly controlled patients with diabetes. Implemented QI projects and patient education documents.
Four rural FQHCs in Indiana^16^	2	2 years	P2 and P3 onsite 12 total hours per week	About 42–45% of patients are enrolled in Medicaid and/or Medicare and 22–23% of patients are uninsured. About 10% of the clinics' patients are native Spanish speakers.	Students were responsible for completing PAs, coordinating MAP applications, tracking contraceptive device prescription processing, and managing inventory of medication samples at the main clinic. Students also performed follow-up phone calls with patients and pharmacies as needed expedite medication processing and patient care activities.	In one year, over 2000 tasks were documented. Due to the workload, the program expanded the next year to 16 h per week. Final interviews showed students enjoyed the opportunity to work with vulnerable and underserved populations. Students got firsthand experience in appropriate written communication, thorough documentation, interprofessional collaboration with providers to address insurance and/or patient barriers, and dissemination of clinical information to insurance companies to justify a medication's necessity. Students collaborated on poster presentations related to the internship program.

Key:

FQHC – federally qualified health center.

PharmD – Doctor of Pharmacy.

P2 – second professional year student pharmacist.

P3 – third professional year student pharmacist.

P4 – fourth professional year student pharmacist.

MAP – medication assistance program.

PA – prior authorizations.

Kadakia et al. (2022) described an FQHC rotation in Indiana where fourth year student pharmacists submitted reflections about their experiences in FQHCs. The 43 reflections from 11 students showed that students advanced their communication skills, collaboration, and empathy.^17^ While these data appear promising for the benefit of trainees, they have limitations as students may have been influenced to acquiesce their responses to be socially desirable as their preceptors would read them. Additionally, there were no objective data to compare the student reflections to measure growth in communication, collaboration, and empathy. Finally, there was no discussion of direct benefit to patients by having students involved in their care described, for example, patient satisfaction, improved clinical measures, or increased medication access.

Scoular et al. (2020) describes another student pharmacist experience with underserved populations occurred with an international Advanced Pharmacy Practice Experience (APPE). Twelve fourth year student pharmacists completed a 6-week international rotation in rural Guatemala with virtual distance precepting between September 2015 and April 2019. In this experience, student pharmacists spent 60–70% of their time in patient-facing activities such as medication counseling, community nurse visits, and collaborating with the physician team that consisted of family medicine residents from the United States and local Guatemalan physicians. Students made 1191 patient care recommendations and 81.4% were accepted by the medical team.^19^ Additionally, students initiated quality improvement projects onsite. This demonstrates tangible evidence that there was a direct impact on patient care via clinical consultations, recommendations, and quality assurance projects. Students also performed weekly reflections. General themes from these reflections included that students felt they gained skills they would not have received elsewhere, became more aware of the needs of diverse patients, and communicated via multiple strategies with patients and the healthcare team. This further exemplifies what has been previously published regarding medical students rotating at international sites and the improvement of clinical skills, compassion and sensitivity to patient needs, and clarity regarding their career goals.^20^, ^21^, ^22^ All students successfully completed the rotation and the preceptors did not have concerns with knowledge base or professionalism; however, the authors mentioned that students must self-select this opportunity and pay additional travel fees. Thus, it would be expected that the students are receptive to the distinct international and virtual preceptor rotation environment. Both Scoular and Owens' studies have shown an improvement in the student's appreciation of cultural diversity and public health in underserved populations.^19^^,^^23^

Payne et al. (2018) describes a pre-post intervention among 68 third and fourth year pharmacy students who completed a clinical rotation at a Colorado FQHC; however, only 37 students completed both the pre and post assessments for full analysis. Student activities on rotation included medication counseling, medication device teaching, and chronic disease state education and management.^24^ These authors concluded that clinical rotations within an FQHC can positively impact pharmacy students' attitudes towards underserved populations. Seven of the eighteen survey questions showed a statistically significant change from baseline in domains such as personal impact, perceptions/ barriers, and educational satisfaction. About 92% of students felt that the FQHC rotation provided more information related to underserved populations to their previous experiences.^24^ Additionally, 59% of students felt they could pursue a job as a pharmacist providing care to underserved populations.^24^ While this surveyed population was small, it does provide objective data that students perceptions about caring for FQHC patients changed within a 6-week period in seven key areas (i.e., working with underserved patients is very satisfying, I feel that I can significantly impact the healthcare of the underserved through pharmacy services, Underserved patients are more likely to accept my recommendation than other patient populations, I would like to work with underserved populations in my pharmacy career, pharmacy education should include exposure to underserved patient care, I feel that I have enough exposure to underserved populations in my pharmacy training, and I feel that my pharmacy education adequately prepared me to provide care to underserved patients).

Simmons et al. (2019) describes a model of a student-run diabetes education and management clinic in a Texas FQHC. The manuscript title and premise have concerning elements as students are not licensed as independent practitioners. The verbiage used to describe the service may have been selected to attract attention as a novel approach to providing care for patients in FQHCs. Additionally, this study asserted that one of the goals of the clinic is “to integrate student clinical experiences and faculty practice in effective delivery of health care services.” We would argue that those items are not an FQHC goal, but rather a means to expand access to diabetes education in the hopes of improving patient care, patient outcomes, and HRSA measures. Simmons describes the success of the student-led service due to highly motivated pharmacy students, but this factor cannot be guaranteed by every individual or cohort that rotates through FQHCs nationally. While student evaluations of the experience were positive, there is a lack of data related to impact on patient care or increasing the quality-of-care received by students versus standard of care with the primary care provider or with a pharmacist-led visit.

In contrast, Fenn et al. (2019) describe a novel approach to integrating student pharmacists into FQHCs over a two-year paid internship. This takes into consideration transitions and turnover of one and continuity of another ensuring at least one intern has had experience as the FQHC intern. This model integrated a second- and third-year (P2 and P3) pharmacy student into FQHCs in Indiana to assist with MAP applications, PAs, medication sample procurement, and contraception inventory management.^17^ Additionally, students were precepted by a PGY1-trained pharmacist completing their post-graduate fellowship. Students completed over 2000 tasks during a one-year period working a total of 12 h per week. Students were able to apply their knowledge of the PA process and MAP processes to expand access to medications for FQHC patients. This assisted the FQHC staff in task delegation and had objective outcomes such as the number of MAP applications, and PAs processed annually, at 208 and 1582 respectively.^18^ These data show direct impact to patient care in a sustainable manner given the students work longitudinally over a two-year period with the P2 student staying on for their P3 year for continuity to train the incoming P2 student. Of the previously discussed FQHC student rotation experiences, this integration appears to be the most impactful, sustainable, and lowest risk of harm to patient care.

## 5 Limited evidence of trainee impact on patient care

FQHC reliance on a trainee labor force may allow greater access to healthcare that would not have been possible otherwise given economic constraints. However, the clinical impact of using trainee labor requires more study. An observational, retrospective study by Choi et al. (2023) analyzed all 1373 FQHCs reported to the UDS system for clinical and nonclinical outcomes of FQHCs with medical training programs compared to those that did not have medical training programs. The investigators found sites that provided medical training programs were associated with an increase in various immunization, screening, and medication use rates. These data do not, however, address the impact of a more than 2:1 ratio of training FQHCs to non-training FQHCs or the impact of a mean difference of 152 employees in favor of training FQHCs.^25^ This makes it difficult to tell if the increase in these rates was due to the quality of care or the larger workforce.

Similarly, a study by Nagelkerk et al. (2018) analyzed diabetes health outcomes associated with an interprofessional education program of medical, pharmacy, and physician assistant students at an FQHC. Pre- and post-data of lab values, patients seen per hour, numbers of physical exams, and patient satisfaction were evaluated. The interprofessional program resulted in decreased body mass index, total cholesterol to high-density lipoprotein ratio, and triglycerides for the patients. The study also found an increase in mean number of patients seen per hour, foot exams performed, and patient satisfaction scores.^26^ The study had no control comparator of a cohort at the same clinic seen by fully licensed professionals and did not address the quality of care delivered by trainees.

## 6 Summary of key points

After reviewing the literature, there are many ways that pharmacists are integrated in patient care at FQHCs whether it be a dispensing role, medication procurement, or disease state management role. Patients have a preference for provider congruence and the ability to communicate directly with their provider versus use of interpretation services. Many FQHC pharmacists will serve as preceptors for schools of pharmacy where they will precept students on their experiential rotations. The data show students learn and grow in clinical experiences at FQHCs and clinics that provide services to underserved or vulnerable populations. However, there are limited data regarding whether patients benefit from interacting with students at FQHCs. While student pharmacists will continue to rotate at FQHC sites, schools of pharmacies and pharmacist preceptors must be diligent to ensure patient safety and satisfaction are at the forefront. Therefore, we will describe strategies to prioritize patient care and reinforce ethical patient care for students who rotate at FQHCs.

## 7 Call to action

Reliance on a primarily trainee workforce prevents patients from receiving the same quality of care as provided in private health centers. As trainees, their experiences and expertise are limited, which has implications for how they provide care, even those who moved into the post-graduate training cycle. While the incoming trainee is likely a newly licensed pharmacist or in pursuit of licensure, they are at the beginning of their post graduate training program. There are also varying levels of trainee experiences such as APPE student pharmacists, postgraduate year 1 or 2 residents, and fellows. There is even variance in the baseline knowledge of individual trainees with the same amount of experience. Patients in FQHCs report frustration with continuity of care because they receive care from a new pharmacy trainee each appointment.^16^ Lastly, the time spent supervising and precepting distracts from time that could otherwise be dedicated to patient care. Supervision also contributes to longer appointment times which can impact patients' access to bus schedules, timely returning to their jobs, childcare arrangements, etc.

Healthcare professionals and administrators must reimagine the FQHC workflow and training practices with the intention of optimizing patient care as the driving force of decision making. FQHCs primary purpose should not be that of a training ground. Patients must be the focus of FQHC operations as FQHC practitioners are visitors in these communities and were designed to serve patients - not the reverse. Therefore, we assert a patient-centered approach to FQHC operations includes the following:

### 7.1 Consider reducing or eliminating FQHCs reliance on trainee labor

The economics of FQHC operation have been a driving force for the current model of their use of trainee labor to provide patient care. At a minimum, the status quo by and large requires the most vulnerable patients see a medical provider with a lower level of training than their adequately insured and resourced counterparts at private clinics. If patient care was centered in FQHC operations, patients would have more time with providers who have more training, experience, and credentials. Using trainees as force multiplier extensions of the precepting pharmacist may sacrifice patient care quality.

### 7.2 More direct oversight of trainees for a longer period by precepting pharmacists

Given the extent to which FQHCs depend on trainee labor, the optimal scenario of not relying on them for operation at all may not be feasible. If FQHCs must continue their reliance on trainee labor to provide patient care, there must be more supervision. Learning to practice independently is a goal of training, but it cannot be at a detriment to patients. Incorporating trainees into the workflow without adequate supervision can lead to subpar delivery of services such as sliding scale fee implementation, 340B pharmacy dispensing, and MAP enrollment due to the natural learning curve of all learners. Trainees are also less likely to communicate errors and have a lower understanding of how operations impact patient safety than fully trained staff.^27^ Trust in a provider is crucial to the success and completeness of patient care.^28^ Previous research suggests that patients are often willing to accept the involvement of a trainee but there is an underlying theme of them doing so on the condition that a preceptor is present and that the trainee's role is supporting the precepting provider.^26^^,^^29^, ^30^, ^31^, ^32^ Until the trainee can operate within the workflow independently for their specific role, they should have consistent, direct supervision by a precepting pharmacist or a post graduate trainee who can operate independently in the precepting pharmacist role.

### 7.3 Shift from economically focused metrics to patient-focused metrics

While the ability to provide care to more patients is positive, the “at least they have care of some sort” approach is neither just nor humane. Patients seen per hour, visits per month, or relative value units as metrics do not describe the quality of care – only how fast providers are in-and-out of patient rooms. Productivity metrics as a primary means of assessing healthcare providers reinforces the economic driver of FQHC operation. Using Press Ganey surveys and comprehensive health outcomes metrics focused on the patient's experience and health may lead to better patient care.^33^^,^^34^ Press Ganey surveys are administered to patients of healthcare organizations to elicit feedback on their experiences with medical providers and the healthcare system. These data are then collated and shared with the healthcare system to help improve the quality of patient care and patient satisfaction.

### 7.4 Emphasizing FQHC patients' humanity during training

There are many indignities that occur when patients interact with the healthcare system, particularly for marginalized patients.^35^ These patients often don't have options to get their care elsewhere because of the lack of private health centers in low-income areas that are currently served by FQHCs. Cultural humility training should be required by FQHC training sites for all trainees and employees prior to them engaging in direct patient care.^36^ Instances of using concerning or racist language towards patients, inappropriate displays of empathy, misgendering trans patients, and approaching patients as “interesting cases” or curiosities rather than living humans are just some of the issues the authors have experienced with trainees in FQHC spaces.

### 7.5 Increased economic support for FQHC operations

Funding these public healthcare centers should be of at least equal political concern as the funding of public education, public housing, public transportation, and public security. Recent surveys of national priorities among Americans rank healthcare availability among the most immediate issues to be addressed in the United States. A 2022 Pew Research Center poll found that “reducing healthcare costs” was the second highest priority among their sample of 5128 adults only behind “strengthening the economy.”^37^ A 2021 poll from the Center for American Progress of 2000 registered voters found that 65% of respondents felt that providing healthcare for financially struggling Americans was more important than addressing budget deficits by decreasing government assistance for low-income Americans. This was the predominant sentiment regardless of political party affiliation and was more common in millennial and generation Z voters.^38^ This suggests that Americans see investing in the nation's healthcare infrastructure for those who need it as a worthwhile priority that ought to be more heavily funded.

## 8 Conclusion

FQHCs provide care and expand healthcare access to underserved and vulnerable patient populations. Pharmacists are often integrated in interprofessional and collaborative practice models at FQHCs via CDTM and consult services that may also involve pharmacy trainees. This places a greater responsibility on the pharmacist preceptor to ensure that patients are receiving high quality evidence-based care. While pharmacy trainees may benefit from learning and training at FQHCs, the learning should not be at the expense of patient care. Pharmacist preceptors may need to provide more direct supervision, emphasize patient humanity, and intervene or even remove trainees who put patient safety at risk. Patients who utilize FQHCs for primary care services deserve to have competent and compassionate clinicians advocating for them to enhance their health status and outcomes.

## Credit author statement

NR and JW contributed to the conceptualization and writing of the manuscript. EDS was consulted for themes and background context and reviewed the manuscript with feedback prior to submission.

## Declaration of Competing Interest

The authors declare that they have no known competing financial interests or personal relationships that could have appeared to influence the work reported in this paper.
